# 2-(4-Fluoro­phen­yl)-2-oxoethyl 2-methoxy­benzoate

**DOI:** 10.1107/S1600536812002577

**Published:** 2012-01-25

**Authors:** Arun M. Isloor, B. Garudachari, M. N. Satyanarayan, Thomas Gerber, Eric Hosten, Richard Betz

**Affiliations:** a National Institute of Technology-Karnataka, Department of Chemistry – Organic Electronics Division, Surathkal, Mangalore 575 025, India; b National Institute of Technology-Karnataka, Department of Physics, Surathkal, Mangalore 575 025, India; c Nelson Mandela Metropolitan University, Summerstrand Campus, Department of Chemistry, University Way, Summerstrand, PO Box 77000, Port Elizabeth 6031, South Africa

## Abstract

In the title compound, C_16_H_13_FO_4_, the aromatic rings enclose an angle of 73.68 (6)°. In the crystal, C—H⋯O and C—H⋯F contacts connect the mol­ecules into a three-dimensional network. The shortest inter­centroid distance between two aromatic π-systems is 3.6679 (7) Å and is apparent between the fluorinated phenyl groups.

## Related literature

For general background to photosensitive protective groups and their synthetic potential, see: Sheehan & Umezaw (1973 ▶); Ruzicka *et al.* (2002 ▶); Litera *et al.* (2006 ▶); Rather & Reid (1919 ▶); Huang *et al.* (1996 ▶); Gandhi *et al.* (1995 ▶). For the graph-set analysis of hydrogen bonds, see: Etter *et al.* (1990 ▶); Bernstein *et al.* (1995 ▶).
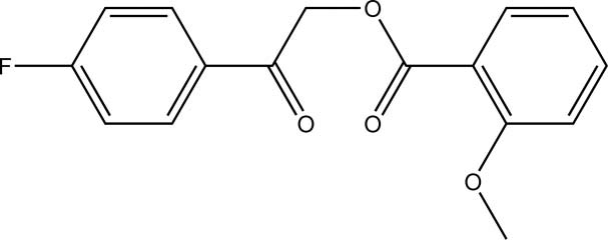


## Experimental

### 

#### Crystal data

C_16_H_13_FO_4_
*M* *_r_* = 288.26
Monoclinic, 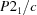

*a* = 7.9370 (3) Å
*b* = 26.4456 (9) Å
*c* = 7.0635 (2) Å
β = 113.404 (1)°
*V* = 1360.64 (8) Å^3^
*Z* = 4
Mo *K*α radiation
μ = 0.11 mm^−1^
*T* = 200 K
0.43 × 0.32 × 0.27 mm

#### Data collection

Bruker APEXII CCD diffractometer
Absorption correction: multi-scan (*SADABS*; Bruker, 2008 ▶) *T*_min_ = 0.682, *T*_max_ = 0.746
12541 measured reflections
3365 independent reflections
2927 reflections with *I* > 2σ(*I*)
*R*_int_ = 0.027

#### Refinement

*R*[*F* ^2^ > 2σ(*F* ^2^)] = 0.037
*wR*(*F* ^2^) = 0.098
*S* = 1.04
3365 reflections
191 parameters
H-atom parameters constrained
Δρ_max_ = 0.27 e Å^−3^
Δρ_min_ = −0.16 e Å^−3^

### 

Data collection: *APEX2* (Bruker, 2010 ▶); cell refinement: *SAINT* (Bruker, 2010 ▶); data reduction: *SAINT*; program(s) used to solve structure: *SHELXS97* (Sheldrick, 2008 ▶); program(s) used to refine structure: *SHELXL97* (Sheldrick, 2008 ▶); molecular graphics: *ORTEP-3* (Farrugia, 1997 ▶) and *Mercury* (Macrae *et al.*, 2008 ▶); software used to prepare material for publication: *SHELXL97* and *PLATON* (Spek, 2009 ▶).

## Supplementary Material

Crystal structure: contains datablock(s) I, global. DOI: 10.1107/S1600536812002577/gk2452sup1.cif

Structure factors: contains datablock(s) I. DOI: 10.1107/S1600536812002577/gk2452Isup2.hkl

Supplementary material file. DOI: 10.1107/S1600536812002577/gk2452Isup3.cdx

Supplementary material file. DOI: 10.1107/S1600536812002577/gk2452Isup4.cml

Additional supplementary materials:  crystallographic information; 3D view; checkCIF report

## Figures and Tables

**Table 1:** Hydrogen-bond geometry (Å, °)

*D*—H⋯*A*	*D*—H	H⋯*A*	*D*⋯*A*	*D*—H⋯*A*
C16—H16⋯F1^i^	0.95	2.52	3.4335 (15)	162
C23—H23⋯O1^ii^	0.95	2.53	3.4004 (15)	152
C25—H25⋯O3^iii^	0.95	2.34	3.1436 (14)	142

Symmetry codes: (i) 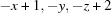; (ii) 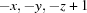; (iii) 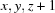.

**Fig. 1.**
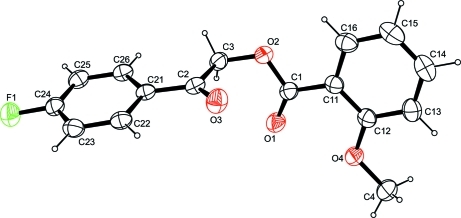
The molecular structure of the title compound, with atom labels and anisotropic displacement ellipsoids drawn at the 50% probability level.

**Fig. 2.**
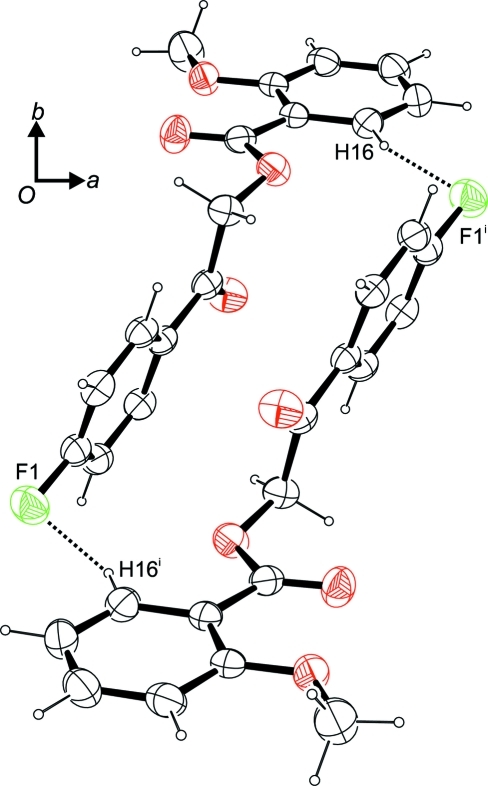
Intermolecular contacts viewed along [0 0 - 1]. For clarity, only the intermolecular C–H···F contacts are depicted. Symmetry operator: (i) -x + 1, -y, -z + 2.

**Fig. 3.**
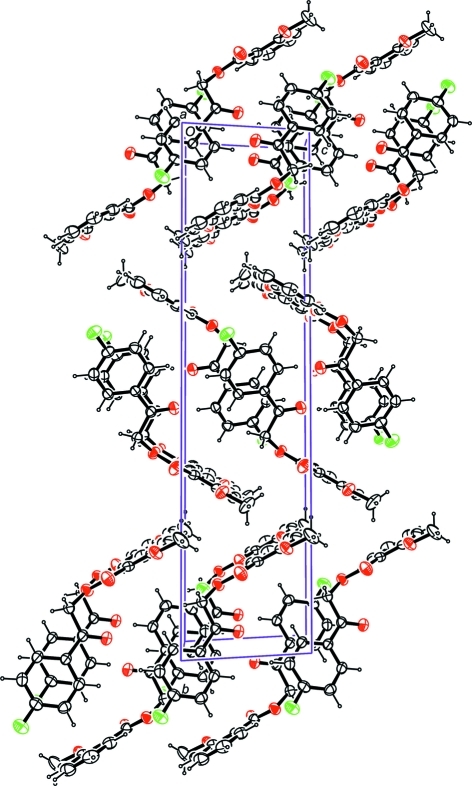
Molecular packing of the title compound viewed along [-1 0 0] (anisotropic displacement ellipsoids drawn at the 50% probability level).

## supplementary crystallographic information

### Comment

Phenacyl benzoates are very useful protecting groups which can easily be cleaved by non-chemical methods. The advantage of photosensitive blocking groups in general is that they can be removed under completely neutral and mild conditions (Sheehan & Umezaw, 1973; Ruzicka *et al.*, 2002; Litera *et al.*, 2006) and therefore used for the identification of organic acids (Rather & Reid, 1919), synthesis of oxazoles, imidazoles (Huang *et al.*, 1996) and benzoxazepine (Gandhi *et al.*, 1995). Keeping this in view, the title compound was synthesized to study its crystal structure.

The title compound is the ester derived of 2-methoxybenzoic acid and 1-(4-fluorophenyl)-2-hydroxyethanone. The least-squares planes defined by the carbon atoms of the two individual aromatic systems intersect at an angle of 73.68 (6)°. The dihedral angle defined by the CO atoms of both carbonyl groups was found at more than 102° (Fig. 1).

In the crystal, C–H···O contacts as well as C–H···F contacts whose range falls invariably by more than 0.1 Å below the sum of van-der-Waals radii of the respective atoms are present. The C–H···O contacts are apparent between both hydrogen atoms in *ortho* position to the fluorine atom on the phenyl group and have the two different carbonyl groups as acceptors. The C–H···F contact is supported by the hydrogen atom in *ortho* position to the carboxylic acid group on the second aromatic system. Details about metrical parameters as well as information about the symmetry of these contacts is summarized in Table 1. In terms of graph-set analysis (Etter *et al.*, 1990; Bernstein *et al.*, 1995), the descriptor for the C–H···O contacts is *C*^1^_1_(6)*R*^2^_2_(18) on the unitary level while the C–H···F contacts require a *R*^2^_2_(24) descriptor on the same level. In total, the molecules are connected to a three-dimensional network. The shortest intercentroid distance between two π-systems was measured at 3.6679 (7) Å and is apparent between the fluorinated phenyl groups (Fig. 2).

The packing of the title compound in the crystal structure is shown in Figure 3.

### Experimental

A mixture of 2-methoxybenzoic acid (1.0 g, 0.0065 mol) potassium carbonate (0.99 g, 0.0072 mol) and 2-bromo-1-(4-fluorophenyl)ethanone (1.41 g, 0.0065 mol) in dimethylformamide (10 ml) was stirred at room temperature for 2 h. On cooling, colorless needle-shaped crystals of 2-(4-fluorophenyl)-2-oxoethyl 2-methoxybenzoate began to separate. These were collected by filtration and recrystallized from ethanol. Yield: 1.62 g, 85.7% (m.p. 359–360 K).

### Refinement

Carbon-bound H atoms were placed in calculated positions (C—H 0.95 Å for aromatic carbon atoms and C—H 0.99 Å for methylene groups) and were included in the refinement in the riding model approximation, with *U*(H) set to 1.2*U*_eq_(C). The H atoms of the methyl groups were allowed to rotate with a fixed angle around the C—C bond (C—H 0.98 Å) to best fit the experimental electron density (HFIX 137 in the *SHELX* program suite (Sheldrick, 2008)), with *U*(H) set to 1.5*U*_eq_(C).

### Crystal data

**Table:**

C_16_H_13_FO_4_	*F*(000) = 600
*M**_r_* = 288.26	*D*_x_ = 1.407 Mg m^−^^3^
Monoclinic, *P*2_1_/*c*	Melting point = 359–360 K
Hall symbol: -P 2ybc	Mo *K*α radiation, λ = 0.71073 Å
*a* = 7.9370 (3) Å	Cell parameters from 8158 reflections
*b* = 26.4456 (9) Å	θ = 2.3–28.3°
*c* = 7.0635 (2) Å	µ = 0.11 mm^−^^1^
β = 113.404 (1)°	*T* = 200 K
*V* = 1360.64 (8) Å^3^	Platelet, colourless
*Z* = 4	0.43 × 0.32 × 0.27 mm

### Data collection

**Table:**

Bruker APEXII CCD diffractometer	3365 independent reflections
Radiation source: fine-focus sealed tube	2927 reflections with *I* > 2σ(*I*)
graphite	*R*_int_ = 0.027
φ and ω scans	θ_max_ = 28.3°, θ_min_ = 2.8°
Absorption correction: multi-scan (*SADABS*; Bruker, 2008)	*h* = −10→10
*T*_min_ = 0.682, *T*_max_ = 0.746	*k* = −34→35
12541 measured reflections	*l* = −9→9

### Refinement

**Table:**

Refinement on *F*^2^	Primary atom site location: structure-invariant direct methods
Least-squares matrix: full	Secondary atom site location: difference Fourier map
*R*[*F*^2^ > 2σ(*F*^2^)] = 0.037	Hydrogen site location: inferred from neighbouring sites
*wR*(*F*^2^) = 0.098	H-atom parameters constrained
*S* = 1.04	*w* = 1/[σ^2^(*F*_o_^2^) + (0.0429*P*)^2^ + 0.365*P*] where *P* = (*F*_o_^2^ + 2*F*_c_^2^)/3
3365 reflections	(Δ/σ)_max_ < 0.001
191 parameters	Δρ_max_ = 0.27 e Å^−^^3^
0 restraints	Δρ_min_ = −0.16 e Å^−^^3^

### Fractional atomic coordinates and isotropic or equivalent isotropic displacement parameters (Å^2^)

**Table:**

	*x*	*y*	*z*	*U*_iso_*/*U*_eq_	
F1	0.06897 (11)	−0.10183 (3)	1.15257 (13)	0.0497 (2)	
O1	0.16097 (12)	0.14995 (4)	0.54890 (14)	0.0429 (2)	
O2	0.44950 (11)	0.12318 (3)	0.72404 (13)	0.0378 (2)	
O3	0.28771 (14)	0.03775 (4)	0.56649 (13)	0.0438 (2)	
O4	0.09706 (11)	0.19590 (3)	0.19131 (13)	0.0399 (2)	
C1	0.31601 (15)	0.14617 (4)	0.56176 (17)	0.0318 (2)	
C2	0.30526 (15)	0.04439 (4)	0.74269 (16)	0.0307 (2)	
C3	0.38626 (16)	0.09361 (4)	0.85111 (17)	0.0344 (2)	
H3A	0.2919	0.1127	0.8803	0.041*	
H3B	0.4897	0.0863	0.9840	0.041*	
C4	−0.0169 (2)	0.21960 (6)	0.0007 (2)	0.0571 (4)	
H4A	0.0247	0.2544	−0.0012	0.086*	
H4B	−0.1445	0.2198	−0.0123	0.086*	
H4C	−0.0087	0.2007	−0.1148	0.086*	
C11	0.39019 (15)	0.16557 (4)	0.41277 (17)	0.0309 (2)	
C12	0.27767 (15)	0.19075 (4)	0.23052 (18)	0.0317 (2)	
C13	0.35424 (18)	0.20974 (5)	0.0989 (2)	0.0402 (3)	
H13	0.2789	0.2272	−0.0230	0.048*	
C14	0.5388 (2)	0.20346 (5)	0.1442 (2)	0.0473 (3)	
H14	0.5895	0.2170	0.0537	0.057*	
C15	0.65086 (18)	0.17783 (5)	0.3194 (2)	0.0466 (3)	
H15	0.7773	0.1731	0.3484	0.056*	
C16	0.57610 (17)	0.15923 (5)	0.4521 (2)	0.0391 (3)	
H16	0.6530	0.1417	0.5729	0.047*	
C21	0.24735 (14)	0.00579 (4)	0.85746 (16)	0.0282 (2)	
C22	0.15576 (15)	−0.03738 (4)	0.75205 (17)	0.0325 (2)	
H22	0.1341	−0.0415	0.6106	0.039*	
C23	0.09629 (15)	−0.07416 (4)	0.85026 (18)	0.0354 (2)	
H23	0.0339	−0.1035	0.7789	0.043*	
C24	0.13080 (15)	−0.06672 (4)	1.05523 (18)	0.0345 (2)	
C25	0.22246 (16)	−0.02547 (5)	1.16654 (17)	0.0348 (2)	
H25	0.2459	−0.0222	1.3088	0.042*	
C26	0.27973 (15)	0.01127 (4)	1.06578 (16)	0.0313 (2)	
H26	0.3416	0.0405	1.1389	0.038*	

### Atomic displacement parameters (Å^2^)

**Table:**

	*U*^11^	*U*^22^	*U*^33^	*U*^12^	*U*^13^	*U*^23^
F1	0.0500 (5)	0.0474 (4)	0.0545 (5)	−0.0017 (3)	0.0238 (4)	0.0127 (3)
O1	0.0357 (5)	0.0503 (5)	0.0443 (5)	0.0094 (4)	0.0176 (4)	0.0130 (4)
O2	0.0321 (4)	0.0377 (4)	0.0370 (4)	−0.0012 (3)	0.0067 (3)	0.0070 (3)
O3	0.0579 (6)	0.0429 (5)	0.0307 (4)	−0.0036 (4)	0.0179 (4)	−0.0037 (3)
O4	0.0325 (4)	0.0410 (5)	0.0434 (5)	0.0045 (3)	0.0123 (4)	0.0132 (4)
C1	0.0322 (5)	0.0260 (5)	0.0333 (5)	0.0010 (4)	0.0090 (4)	−0.0001 (4)
C2	0.0283 (5)	0.0318 (5)	0.0276 (5)	0.0049 (4)	0.0065 (4)	0.0002 (4)
C3	0.0365 (6)	0.0319 (5)	0.0294 (5)	−0.0006 (4)	0.0074 (4)	0.0018 (4)
C4	0.0409 (7)	0.0620 (9)	0.0594 (8)	0.0071 (6)	0.0105 (6)	0.0307 (7)
C11	0.0312 (5)	0.0239 (5)	0.0368 (5)	−0.0018 (4)	0.0125 (4)	−0.0028 (4)
C12	0.0338 (5)	0.0227 (5)	0.0383 (6)	−0.0021 (4)	0.0140 (4)	−0.0021 (4)
C13	0.0480 (7)	0.0314 (6)	0.0443 (6)	−0.0039 (5)	0.0216 (5)	0.0018 (5)
C14	0.0528 (8)	0.0405 (7)	0.0609 (8)	−0.0084 (6)	0.0355 (7)	−0.0028 (6)
C15	0.0366 (6)	0.0422 (7)	0.0670 (9)	−0.0050 (5)	0.0270 (6)	−0.0084 (6)
C16	0.0329 (6)	0.0336 (6)	0.0484 (7)	0.0003 (4)	0.0134 (5)	−0.0029 (5)
C21	0.0251 (5)	0.0292 (5)	0.0271 (5)	0.0044 (4)	0.0070 (4)	−0.0017 (4)
C22	0.0306 (5)	0.0348 (5)	0.0281 (5)	0.0018 (4)	0.0074 (4)	−0.0046 (4)
C23	0.0302 (5)	0.0323 (5)	0.0389 (6)	0.0000 (4)	0.0085 (4)	−0.0040 (4)
C24	0.0293 (5)	0.0339 (6)	0.0399 (6)	0.0056 (4)	0.0134 (4)	0.0073 (4)
C25	0.0353 (6)	0.0403 (6)	0.0281 (5)	0.0069 (5)	0.0118 (4)	0.0004 (4)
C26	0.0306 (5)	0.0310 (5)	0.0286 (5)	0.0035 (4)	0.0079 (4)	−0.0045 (4)

### Geometric parameters (Å, °)

**Table:**

F1—C24	1.3577 (13)	C13—C14	1.3799 (19)
O1—C1	1.2015 (14)	C13—H13	0.9500
O2—C1	1.3571 (13)	C14—C15	1.381 (2)
O2—C3	1.4236 (14)	C14—H14	0.9500
O3—C2	1.2090 (14)	C15—C16	1.3834 (19)
O4—C12	1.3545 (14)	C15—H15	0.9500
O4—C4	1.4339 (15)	C16—H16	0.9500
C1—C11	1.4865 (16)	C21—C26	1.3971 (14)
C2—C21	1.4853 (15)	C21—C22	1.3988 (15)
C2—C3	1.5177 (15)	C22—C23	1.3820 (17)
C3—H3A	0.9900	C22—H22	0.9500
C3—H3B	0.9900	C23—C24	1.3765 (17)
C4—H4A	0.9800	C23—H23	0.9500
C4—H4B	0.9800	C24—C25	1.3722 (17)
C4—H4C	0.9800	C25—C26	1.3835 (17)
C11—C16	1.3990 (16)	C25—H25	0.9500
C11—C12	1.4089 (15)	C26—H26	0.9500
C12—C13	1.3906 (16)		
			
C1—O2—C3	115.22 (9)	C12—C13—H13	119.8
C12—O4—C4	117.22 (10)	C13—C14—C15	120.87 (12)
O1—C1—O2	122.26 (11)	C13—C14—H14	119.6
O1—C1—C11	126.84 (10)	C15—C14—H14	119.6
O2—C1—C11	110.90 (9)	C14—C15—C16	119.00 (12)
O3—C2—C21	121.88 (10)	C14—C15—H15	120.5
O3—C2—C3	119.77 (10)	C16—C15—H15	120.5
C21—C2—C3	118.35 (9)	C15—C16—C11	121.69 (12)
O2—C3—C2	109.74 (9)	C15—C16—H16	119.2
O2—C3—H3A	109.7	C11—C16—H16	119.2
C2—C3—H3A	109.7	C26—C21—C22	118.98 (10)
O2—C3—H3B	109.7	C26—C21—C2	122.43 (10)
C2—C3—H3B	109.7	C22—C21—C2	118.59 (9)
H3A—C3—H3B	108.2	C23—C22—C21	121.07 (10)
O4—C4—H4A	109.5	C23—C22—H22	119.5
O4—C4—H4B	109.5	C21—C22—H22	119.5
H4A—C4—H4B	109.5	C24—C23—C22	117.55 (11)
O4—C4—H4C	109.5	C24—C23—H23	121.2
H4A—C4—H4C	109.5	C22—C23—H23	121.2
H4B—C4—H4C	109.5	F1—C24—C25	118.00 (11)
C16—C11—C12	118.28 (11)	F1—C24—C23	118.29 (11)
C16—C11—C1	120.09 (10)	C25—C24—C23	123.70 (11)
C12—C11—C1	121.63 (10)	C24—C25—C26	118.12 (10)
O4—C12—C13	122.34 (11)	C24—C25—H25	120.9
O4—C12—C11	118.01 (10)	C26—C25—H25	120.9
C13—C12—C11	119.65 (11)	C25—C26—C21	120.56 (10)
C14—C13—C12	120.49 (12)	C25—C26—H26	119.7
C14—C13—H13	119.8	C21—C26—H26	119.7
			
C3—O2—C1—O1	14.38 (15)	C13—C14—C15—C16	−1.2 (2)
C3—O2—C1—C11	−166.53 (9)	C14—C15—C16—C11	0.23 (19)
C1—O2—C3—C2	75.18 (12)	C12—C11—C16—C15	1.31 (17)
O3—C2—C3—O2	−6.54 (15)	C1—C11—C16—C15	−178.44 (11)
C21—C2—C3—O2	174.23 (9)	O3—C2—C21—C26	174.92 (11)
O1—C1—C11—C16	178.44 (11)	C3—C2—C21—C26	−5.87 (15)
O2—C1—C11—C16	−0.61 (14)	O3—C2—C21—C22	−5.59 (16)
O1—C1—C11—C12	−1.31 (18)	C3—C2—C21—C22	173.62 (10)
O2—C1—C11—C12	179.65 (9)	C26—C21—C22—C23	0.49 (16)
C4—O4—C12—C13	3.34 (17)	C2—C21—C22—C23	−179.02 (10)
C4—O4—C12—C11	−177.26 (11)	C21—C22—C23—C24	−0.10 (16)
C16—C11—C12—O4	178.72 (10)	C22—C23—C24—F1	178.27 (10)
C1—C11—C12—O4	−1.53 (15)	C22—C23—C24—C25	−0.94 (17)
C16—C11—C12—C13	−1.87 (16)	F1—C24—C25—C26	−177.68 (10)
C1—C11—C12—C13	177.88 (10)	C23—C24—C25—C26	1.53 (17)
O4—C12—C13—C14	−179.70 (11)	C24—C25—C26—C21	−1.08 (16)
C11—C12—C13—C14	0.91 (17)	C22—C21—C26—C25	0.12 (16)
C12—C13—C14—C15	0.7 (2)	C2—C21—C26—C25	179.61 (10)

### Hydrogen-bond geometry (Å, °)

**Table:**

*D*—H···*A*	*D*—H	H···*A*	*D*···*A*	*D*—H···*A*
C16—H16···F1^i^	0.95	2.52	3.4335 (15)	162
C23—H23···O1^ii^	0.95	2.53	3.4004 (15)	152
C25—H25···O3^iii^	0.95	2.34	3.1436 (14)	142

Symmetry codes: (i) −*x*+1, −*y*, −*z*+2; (ii) −*x*, −*y*, −*z*+1; (iii) *x*, *y*, *z*+1.
